# Evaluation of Fretting Wear of Contact Surfaces of Composite Material-Aircraft Alloy in Nominally Stationary Joints of Aircraft Structures

**DOI:** 10.3390/ma19204336

**Published:** 2026-10-15

**Authors:** Tareq M. A. Al-Quraan, Andrii Khimko, Oksana Mikosianchyk, Margaryta Khimko, Rudolf Mnatsakanov, Igor Trofimov, Natalia Smetankina, Andrii Kondratiev

**Affiliations:** 1 Department of Technical Sciences, Ma’an College, Al-Balqa Applied University, Al-Salt 19117, Jordan; 2 Department of Air Transport, National University «Kyiv Aviation Institute», 03058 Kyiv, Ukraine; andreykhimko@ukr.net (A.K.); m.khimko@h3ops.com (M.K.); mnatsakanov@ukr.net (R.M.); 3 Department of Applied Mechanics and Materials Engineering, National University «Kyiv Aviation Institute», 03058 Kyiv, Ukraine; oksana.mikos@ukr.net; 4 Department of Ecology, Chemistry and Chemical Technology, National University «Kyiv Aviation Institute», 03058 Kyiv, Ukraine; troffi@ukr.net; 5 Department of Vibration and Thermostability Studies, Anatolii Pidhornyi Institute of Power Machines and Systems of the National Academy of Sciences of Ukraine, Komunalnykiv Str. 2/10, 61046 Kharkiv, Ukraine; nsmet@ipmach.kharkov.ua or nsmetankina@ukr.net; 6 Department of Materials Science and Engineering of Composite Structures, O.M. Beketov National University of Urban Economy in Kharkiv, Chornohlazivska Str. 17, 61002 Kharkiv, Ukraine

**Keywords:** fretting wear, fretting corrosion, CFRP, GFRP, titanium alloy, aluminum alloy, metal–composite interface, tribo-oxidation

## Abstract

The article examines and analyzes the use of composite materials, such as carbon fiber-reinforced plastic (CFRP) and glass fiber-reinforced plastic (GFRP), in new-generation aircraft. Comparative studies were conducted on these composite materials alongside aviation alloys based on aluminum, titanium, and steel. It was determined that contact interfaces in nominally stationary joints suffer significant damage due to vibrational loads—stemming from aircraft engines and normal operations—a phenomenon classified as fretting corrosion. The study found that Al-CFRP/GFRP contact leads to the catastrophic degradation of friction surfaces caused by the formation of hard Al_2_O_3_ oxides. Ti-CFRP/GFRP contact is the most common configuration in new-generation aircraft; notably, the wear resistance of the composite materials is 6–20 times higher than that of the titanium alloy, owing to the inherent properties of titanium and its susceptibility to wear. Furthermore, the article analyzes surface processing and protection methods for titanium components aimed at enhancing the durability and wear resistance of these contact pairs.

## 1. Introduction

Composite materials are one of the most common materials in the aviation industry for the construction of aircraft structures. High specific strength of materials allows for the creation of ultra-light and heavy-duty structures, which are highly in demand in aviation, space and other industries. Modern aircraft use up to 50% of carbon fiber and fiberglass materials in the power structures of wings [1], fuselages and aircraft engines. Titanium and steel cannot be removed from structures due to their physical and mechanical properties, which are not inherent in composite materials. Thus, contacts of carbon fiber (CFRP) and fiberglass (GFRP) materials with metallic materials based on aluminum, titanium and steel are increasingly appearing in modern aircraft and aircraft that were designed after the 2000s. Such contacts are implemented in nodes and connections, which are called nominally stationary [2,3,4] and, as a rule, fix something and are considered by nature as being fixed-detachable.

The factor of vibration loads from aircraft engines and operational loads during flight and maneuvers is the norm for normal aircraft operation [5]. The wear of materials in nominally stationary contacts under the influence of vibration load is associated with the actions of loads that occur during the relative cyclic movement (from 1 to 5 μm) of the contacting joints and is manifested as a deterioration in the quality of surfaces, a decrease in fatigue strength and a decrease in geometric dimensions [6,7,8,9]. The nature and intensity of wear depends on the physical and mechanical properties of the materials in contact, the parameters of mechanical action, and temperature, as well as the chemical activity of the medium and other factors.

Over time, contacts that were considered nominally stationary in aircraft under the influence of vibration begin to move with the occurrence of specific damage to the contacting surfaces, which is called fretting corrosion. A feature and a big problem of fretting corrosion is the formation of microcracks on the surfaces of parts, with their subsequent destruction and loss of contact bond strength [10]. Since some cases of destruction of the contact of composite materials with metal aviation alloys with loss of structural strength as a result of vibration loads [11,12,13] have been recorded, there is a need to analyze and investigate such joints for wear resistance.

The analysis of the application of aviation alloys and polymer composite materials in the aviation industry [1,14] shows their mass use for power structural elements of both aircraft and aircraft engines.

In References [15,16,17] a review and analysis of the development of new composite structures for the wings of An-type aircraft, by the Antonov State Enterprise, is carried out. Composite materials are discussed at the annual international exhibition “Sampe 2019” under the NEWS project, which was formed in accordance with the strategy of “STELIA Aerospace R&T” for the development of composite materials and technologies with high technical and economic potential. The authors of [15], referring to representatives of the Airbus company, note that the main material for the operation of aircraft for 2025–2035 [17] is carbon fiber material (CFRP, CFRTP), which is certified in accordance with EASA and FAA.

The authors of Reference [17] show the massive use of CFRP material for Boeing 737 MAX, 777X, 787, 787 Dreamliner and Airbus A320, A350, A350 XWB, etc. as part of the implementation of Boeing’s NMA (New Midsize Airplane) aircraft project, which represents the next series of aircraft—compared to the Boeing 787, 777X and Airbus A350 already in operation.

Carbon fiber-reinforced polymers, carbon-reinforced plastics, and carbon-reinforced thermoplastics (CFRP, CRP, CFRTP), also known as carbon fiber, carbon composite, or simply carbon, are extremely durable and lightweight fiber-reinforced composite materials containing carbon fibers. CFRPs are commonly used wherever a high strength-to-weight ratio and rigidity are required, such as in aerospace, ship superstructures, automotive, civil engineering, and sports equipment [18,19,20].

The binding polymer is often a thermosetting resin, such as epoxy, but other thermosetting or thermoplastic polymers such as polyester, vinyl ester, or nylon are used [21]. The properties of the carbon fiber final product can be affected by the type of additives introduced into the binding matrix [22]. The most common additive is silicon dioxide, but other additives such as rubber and carbon nanotubes can also be used.

The authors of Reference [23] argue that typical epoxy-based CFRPs exhibit little to no ductility, with a fracture-to-fracture deformation of less than 0.5%. Composite materials with epoxy resin have high strength and modulus of elasticity, while the mechanics of brittle fracture create unique difficulties for engineers in detecting fractures, since failure occurs catastrophically and immediately. Thus, efforts to strengthen carbon CFRPs include modifying the existing epoxy material and finding an alternative polymer matrix. One such promising material is PEEK, which exhibits an order of magnitude of greater viscosity with a similar modulus of elasticity and tensile strength [23]. However, PEEK is much more difficult to process and more expensive.

More than 50% of the materials used in modern aircraft are composite materials, and 45% are carbon fibers, from which power structural elements, aircraft skin panels, spars, fragments of tail unit structures, covers and hatches, engine suspension pylons, engine body nacelles and much more are made [24].

Along with CFRP and CFRTP, fiberglass plastics (GFRP, GFRTP) are also used in aircraft construction. Most often, lighter carbon fiber is used for aircraft, and fiberglass for unloaded parts, panels in the fuselage cockpit, pilot and passenger seats, and nose fairing. Fiberglass is heavier than CFRP and less durable, but it is significantly cheaper. The nose fairing of the aircraft is made of fiberglass, since this part must transmit radio waves, and carbon fiber conducts current and creates interference. All parts are made by vacuum infusion. In terms of its characteristics, it replaces aluminum alloy, but it is not prone to the corrosive effects of the environment. Airbus and Boeing replace aluminum with GFRP in the production of flaps, keels, stabilizers, hatches and doors, window surrounds, and interior elements. This results in reduced fuel operating costs and less need for logistics, which arises when using metals due to their fatigue and corrosion.

Thus, the main composite materials for our tribological studies will be glass fiber-based (GFRP) and carbon fiber-based materials (CFRP). The design of these reinforced fibers may vary, but in 99% of cases, fabrics made from these reinforced fibers are used to create structural elements [25]. The difference is only the number of layers of fabric and the type of weaving of the fabric. The fiber of the composite material can be different in thickness and type of weaving. 1K, 3K, 6K, and 12K are the numbers of filaments of fiber in a single filament to create fabric. 24K and 48K are used in heavy fabrics and tend to have more weight and rigidity. Different types of weaving (one-sided, crosswise, satin, and others) affect the properties of materials such as strength, rigidity, and appearance. In addition, other materials (Kevlar, nylon, copper) are combined with fabrics [26] to increase impact resistance and protection against damage, as well as to affect heat resistance and rigidity.

The analysis of the use of aviation alloys [27,28,29] shows that titanium alloys with the highest strength characteristics are usually used to create power structural elements of aircraft that work in contact with composite materials. For brackets, chassis elements, bolted connections, levers, and shafts of gas turbine engines, strength characteristics are paramount, not temperature resistance and creep. Such materials include, as a rule, such alloys as Ti5Al5V5Mo1Cr1Fe and Ti6Al4V. For example, the Antonov State Enterprise, when designing its aircraft of the AN series, took the material Ti5Al5V5V5Mo1Cr1Fe (VT-22) as a structural titanium alloy used to replace steel parts made of materials 30HGSNA and 30X2VNFA. This alloy is used, for example, to make elements and structural power brackets for An-148, An-72, An-74, An-124, etc., aircraft. Titanium alloys come into contact with composite materials in places such as: landing gear attachment points to the fuselage, joints of tail unit elements, engine attachment points, skin joints (titanium is supplied to avoid corrosion between the composite material and aluminum alloy) and many other places that are subjected to vibration stress during operation.

The wear resistance of composite materials is influenced not only by fibers (carbon fiber or fiberglass), but also by the material of the matrix. Since, first of all, the binder material comes into contact with other materials, and only then is the load transferred to the reinforcing fibers, after the abrasion of the upper layers, the fabric from the reinforcing fibers of composite materials enters the friction process. In addition to various types of matrices, in the production of composite materials, some other elements (dispersion powders, nanotubes) are used to add to the binder to increase the characteristics of strength, heat resistance, electrical conductivity, radio transparency, etc.

On the other hand, the wear resistance of aircraft components can be affected by the aircraft alloy with which the composite material in a pair comes into contact [30,31]. In modern aircraft, the most massive materials are aluminum and titanium alloys, as well as steel. All these materials come into contact with composite materials in certain places in aircraft.

The analysis of the use of GFRP at the Antonov State Enterprise [15,32] showed that the most-used materials for the production of aircraft elements are T-10-14 fiberglass (with a thickness of 0.24 mm) and the following types of composite materials with matrices:

- (has a white color and four layers of T-10-14 fiberglass, production of power structures of the airframe of small aircraft, cabin elements, wing panels);
- 5-211-BN (has a light-yellow color, used for tail panels, helicopter propellers);
- FP-520 (has a dark-red color, used in the electrical circuits of aircraft avionics).

Analysis of aircraft parts based on reinforcing unidirectional fibers CFRP Lup 0.1 [32] showed that the materials for production are the following types of composite materials:

- Carbon 3K plain or VKU 32 (is a unidirectional CFRP, according to its characteristics; it is at the level of Zoltek material and Panex-35-13 or ED-20 filler).
- VKU 33, 35 (used for parts of the airframe’s power structures: wing, center section, mechanization elements, stabilizer skin, spars, forks, integral parts of the wing and fuselage, sound-absorbing structures of gas turbine engines).
- VKU 38 (manufacture of heat-loaded parts and assemblies in high- and medium-loaded aircraft structures: engine body parts, guide blades, inlet guide apparatus of gas turbine engines, and noise absorption panels).
- VKU 38ZHN, VKU 38TR (manufacture of aircraft engine parts; it is operated from minus 60 to plus 300 °C—1000 h and for a short time at a temperature of 330 °C—200 h, layer thickness 0.24–0.28 mm).

Thus, the conducted analysis of literary sources allows us to define the purpose of the study as studying the features of the use of composite materials in modern aircraft structures and assessing the influence of their type on wear resistance when interacting with aircraft alloys under conditions of nominally stationary contacts subject to vibration loads.

## 2. Materials and Methods

Thus, based on the analysis of materials used in aviation and possible zones of their contact with composites, the following groups of materials were selected for research under vibration load conditions:

1. Titanium alloy Ti5Al5V5Mo1Cr1Fe (VT22), which is a structural material for aircraft elements of type An.
2. D16T aluminum alloy (EN AW-2024/EN AW-AlCu4Mg1, AA2024, 2024-T3511, AlCuMg2), the base material for the power structures of aging aircraft.
3. Steel 30CrMnSiA (30HGS, AISI 4130, 14331, 30ChGSA), aviation steel for aircraft power parts.
4. CFRP carbon fiber (Carbon 3K plain with Lup 0.1 fiber and binding ED-20), which is the most popular aviation structural material for all modern aircraft.
5. GFRP fiberglass (with T-10-14 fabric and matrices: EDT-69H, 5-211-BN, FP-520), the material from which some structural elements and elements of aircraft cabins, as well as fuselages of private aviation aircraft, are made.

**Figure 1:**
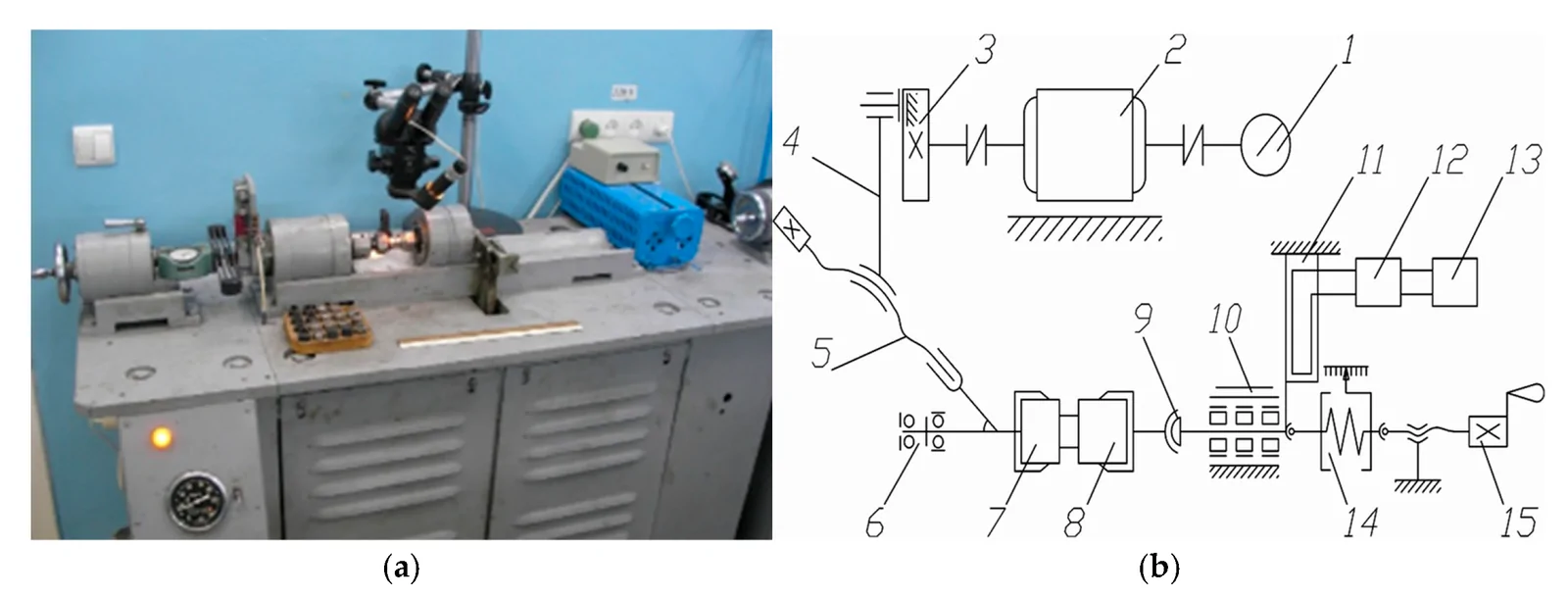
Appearance (**a**) and kinematic diagram (**b**) of the fretting corrosion machine: 1—revolution counter; 2—electric motor; 3—eccentric; 4—vertical connecting rod; 5—tuning device; 6—horizontal connecting rod; 7—movable sample; 8—stationary sample; 9—self-orienting collet; 10—movable headstock; 11—strain gauge; 12—amplifier; 13—recording equipment; 14—dynamometer; and 15—loading device.

The tests were carried out according to the methodology presented in Reference [33]. The essence of the method is that a movable cylindrical sample with a composite material, in contact with an end with a fixed cylindrical sample made of titanium alloys, at a given normal pressure, is driven into rotary-rotational motion with a given amplitude and frequency (Figure 1). The wear of a stationary sample is measured for a given number of cycles, according to the value of which the wear resistance of the test material is determined.

The installation works as follows: the electric motor 2 transmits rotational motion to the eccentric 3 with adjustable eccentricity. The speed and number of revolutions are recorded by the device 1. Eccentric 3 through the connecting rod 4 is connected to the rocker 6 of the shaft 7 of the counter-sample rotary drive 8. Counter-sample displacement amplitude 8 is regulated by an eccentric 3 and a device 5. A fixed sample 9 is fixed in a centered collet 10 mounted on the shaft of a moving headstock 11. The samples are loaded with a dynamometer 14 and a loading device 15. The value of the axial load on the samples is recorded using a dynamometer with a measurement range of 0.2 to 2 kN. Registration of the friction force is carried out by the device 13 through the amplifier 12 using a load bar 11. The number of test cycles was controlled by a meter installed on the front panel of the installation.

The amplitude of oscillations is regulated by changing the eccentricity of the eccentric (roughly) and changing the length of the arm of the horizontal connecting rod (exactly). Coarse amplitude adjustment allows for changing its value from 10 to 1000 microns, accurately—from five to 15 microns. The amplitude of displacement is defined as the difference in the amplitudes of oscillation of moving and stationary samples. The amplitude measurement is carried out directly on the samples using an optical binocular microscope and using the stroboscopic effect.

During wear resistance tests, the coefficient of friction of titanium alloys with composite materials was also recorded. Kinetic changes in the coefficient of friction during the contact of titanium alloys with composite materials make it possible to assess the transient processes of wear of polymer alloys to the contact of titanium alloys with composite materials fibers. For titanium alloys, this means the ability to determine the period of operation of each subsequent layer of different hardnesses on the surface of the alloy.

**Figure 2:**
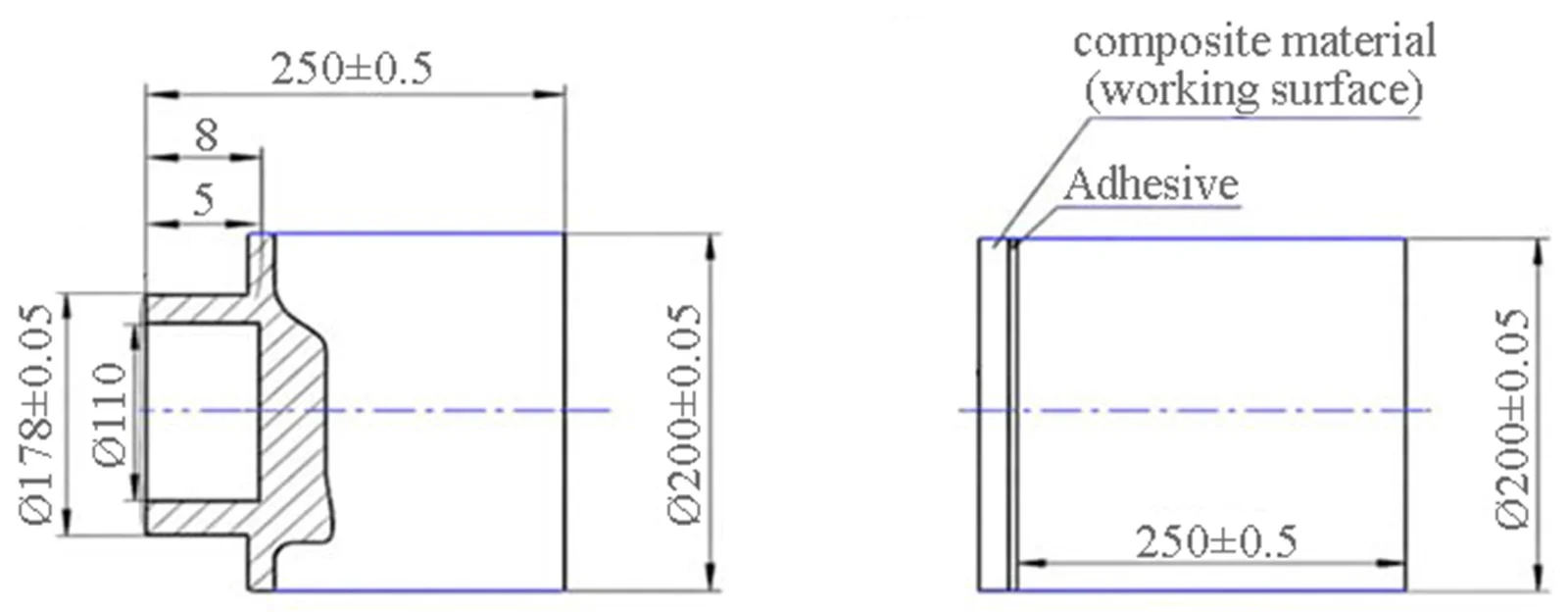
Scheme of the counter sample (**a**) and samples with glued composite material (**b**) for research under vibration loads.

Samples for research (Figure 2) are cylindrical rollers with a diameter of 20 mm, on the end of which plates of composite materials were glued (Figure 2a) from materials Ti5Al5V5Mo1Cr1Fe, AlCu4Mg2 and 30CrMnSiA. Contact occurred on a surface with a nominal contact area of 0.5 cm^2^.

**Figure 3:**
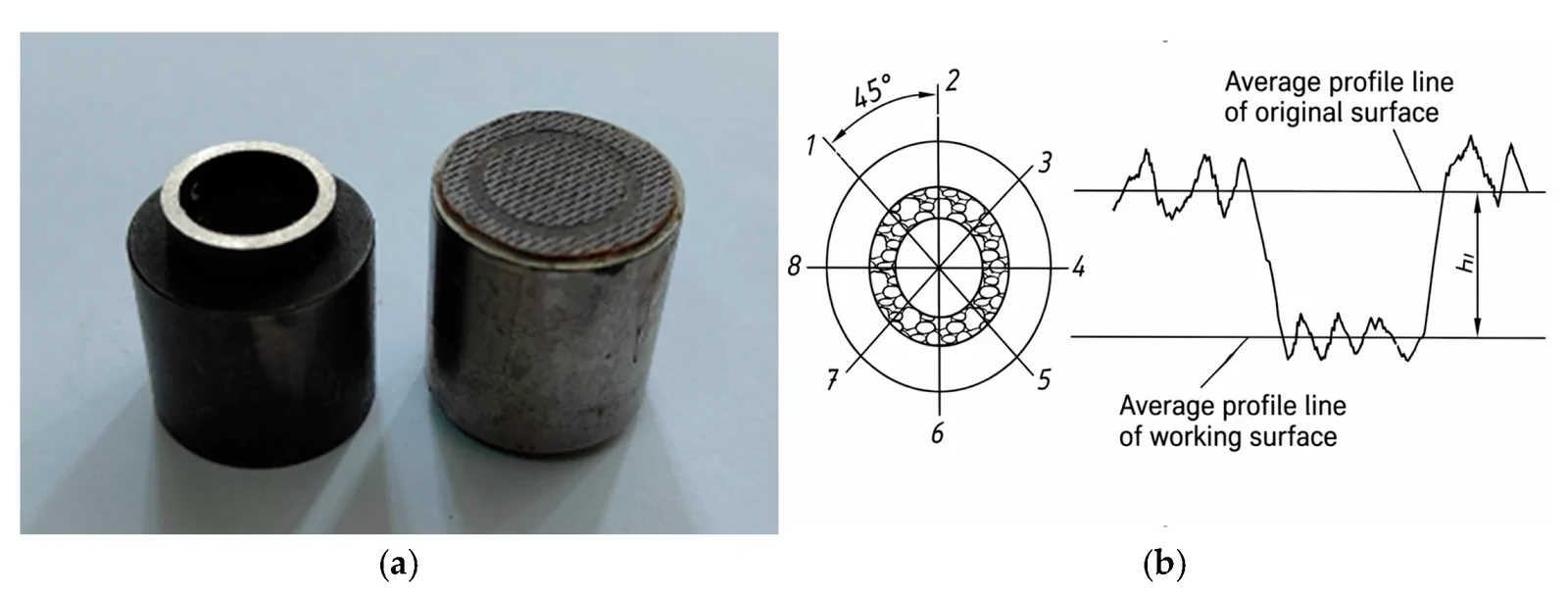
Photos of samples for contact studies (**a**) and wear measurement diagram (**b**) in wear resistance tests of composite materials with aviation alloys: 1-5, 2-6, 3-7, 4-8—directions of lines on the specimen end face after testing, along which linear wear was measured.

The studies were performed with a constant load of 10 MPa and a sample displacement amplitude of 175 μm. The test base was 300 thousand cycles. The frequency of oscillations was 30 Hz, and the test temperature was about 20 °C. Wear resistance studies were carried out without lubricant in air. The samples were wiped with Antisilicone liquid before and after the tests and dried (Figure 3). Wear measurements for a specific section *h_i_* of the specimen were performed using a HANDYSURF 550 instrument (Tokyo Seimitsu Co., Ltd., Tokyo, Japan) in accordance with the ISO 17025:2017 standard [34] by recording profilograms from eight equidistant working surfaces of the specimen in the radial direction; the average wear value was determined as shown in Figure 3b and calculated using Formula (1). According to the value of linear wear, the wear resistance of the studied composite materials was determined. A 95% confidence interval was calculated based on the results of at least three parallel studies using Student’s t-distribution. The relative measurement error for all data points did not exceed 5%. An important advantage of determining wear by the linear method [35] is that the amount of wear does not depend on the specific gravity of the material and possible changes in the mass of the samples.

Linear wear is important in fretting corrosion studies primarily because the contact area and slip amplitudes are microscopic, so the mass loss is so small that it is difficult to measure reliably. In addition, wear products partially remain in the contact zone and on the samples, which distorts mass measurements, and the depth of the wear spot is determined by profilometry based on the displacement of the contact pair. Linear wear is used to calculate wear intensity, specific wear rate, and wear coefficient, i.e., the main tribological parameters that can be compared for different materials and coatings. It also reflects the actual consequences of fretting on the unit (gap growth, loss of tension, redistribution of contact pressures as the wear spot deepens), which is why it is used to evaluate the service life of the connection.

The wear of the sample *h_mid_* is determined by the formula:

(1) $$h_{mid}=\frac{\sum_{1}^{8}h_{i}}{8},$$

where *h_mid_* is the distance on the profile of the friction track between the middle lines of the profile of the source and working surfaces.

## 3. Results

**Figure 4:**
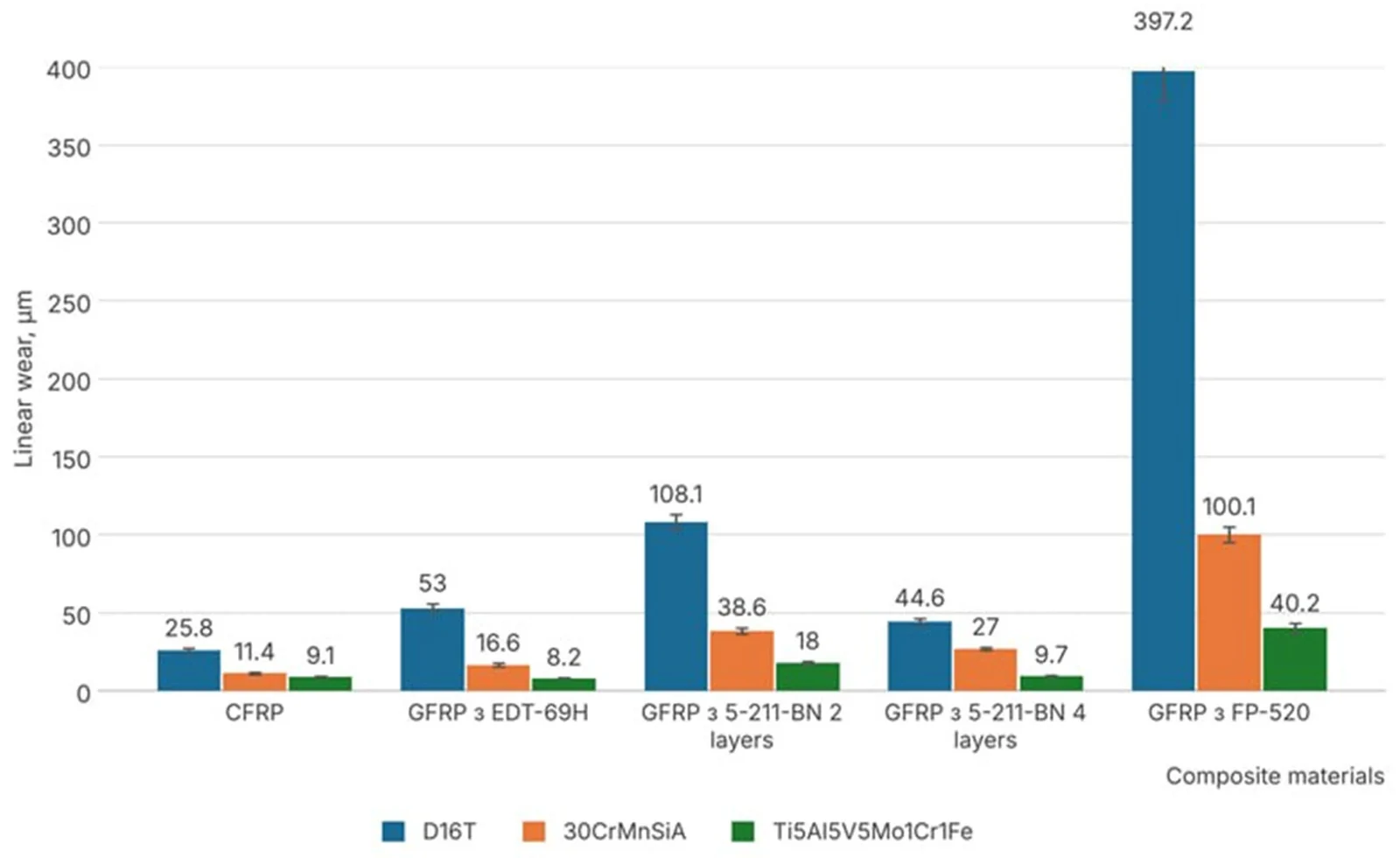
Wear resistance of composite materials with aviation alloys under vibration conditions.

The test results are presented in Figure 4. Analyzing the results of the CFRP/GFRP wear studies, it can be said that the material based on the FP-520 binder has the greatest wear, the vacuum-autoclave molding of which took place at a temperature of 110 ± 5 °C. The main property of this material is low heat generation and reduced smoke generation during combustion, which makes it possible to use it for the manufacture of interior panels of aircraft and other vehicles.

Analyzing the GFRP material with the same 5-211-BN fillers and different layers of fiberglass, we can say that the number of layers of prepreg, which makes up the composite material, significantly affects its wear resistance. In our case, a 2-fold increase in layers provides a 60% increase in strength, which affects the tribological characteristics of the GFRP material with 5-211-BN.

The technological process of composite formation also affects wear resistance. In the case of GFRP material from 5 to 211-BN with two layers of fiberglass, the formation was the same as that of GFRP material with FP-520—vacuum-autoclave molding took place at a temperature of 110 ± 5 °C, affecting wear resistance of the composite material as a whole.

Carbon fibers are more wear-resistant than glass fibers. Although their surface hardness is lower (2–3 on the Mohs scale) than that of glass fiber, they are distinguished by a higher modulus of elasticity (230–700 GPa) and superior abrasion resistance. Despite its good hardness (5.5–6.5 on the Mohs scale) and resistance to certain types of damage, glass fiber has a lower modulus of elasticity (60–90 GPa) compared to carbon fiber (Figure 4) and is more susceptible to mechanical impact. Therefore, in applications requiring high resistance to wear and mechanical loads, carbon fiber is undoubtedly the preferred choice.

It should also be noted that by reinforcing CFRP/GFRP fibers with a binder based on epoxy resins, EDT-69H and ED-20, causes greater wear resistance than with other matrices that were studied during fretting corrosion tests.

When analyzing the contact of CFRP/GFRP with aviation materials, it can be concluded that CFRP/GFRP-AlCu4Mg2 has the greatest damage. Then, with an increase in wear resistance, there is a connection to CFRP/GFRP-30CrMnSiA. The most successful option is the CFRP/GFRP-Ti5Al5V5Mo1Cr1Fe contact. Interestingly, when rubbing with the aluminum alloy AlCu4Mg2, the wear is at a maximum in CFRP/GFRP, which is 2–4 times greater than the wear with 30CrMnSiA steel and 3–10 times greater than the wear with the titanium alloy Ti5Al5V5Mo1Cr1Fe.

**Figure 5:**
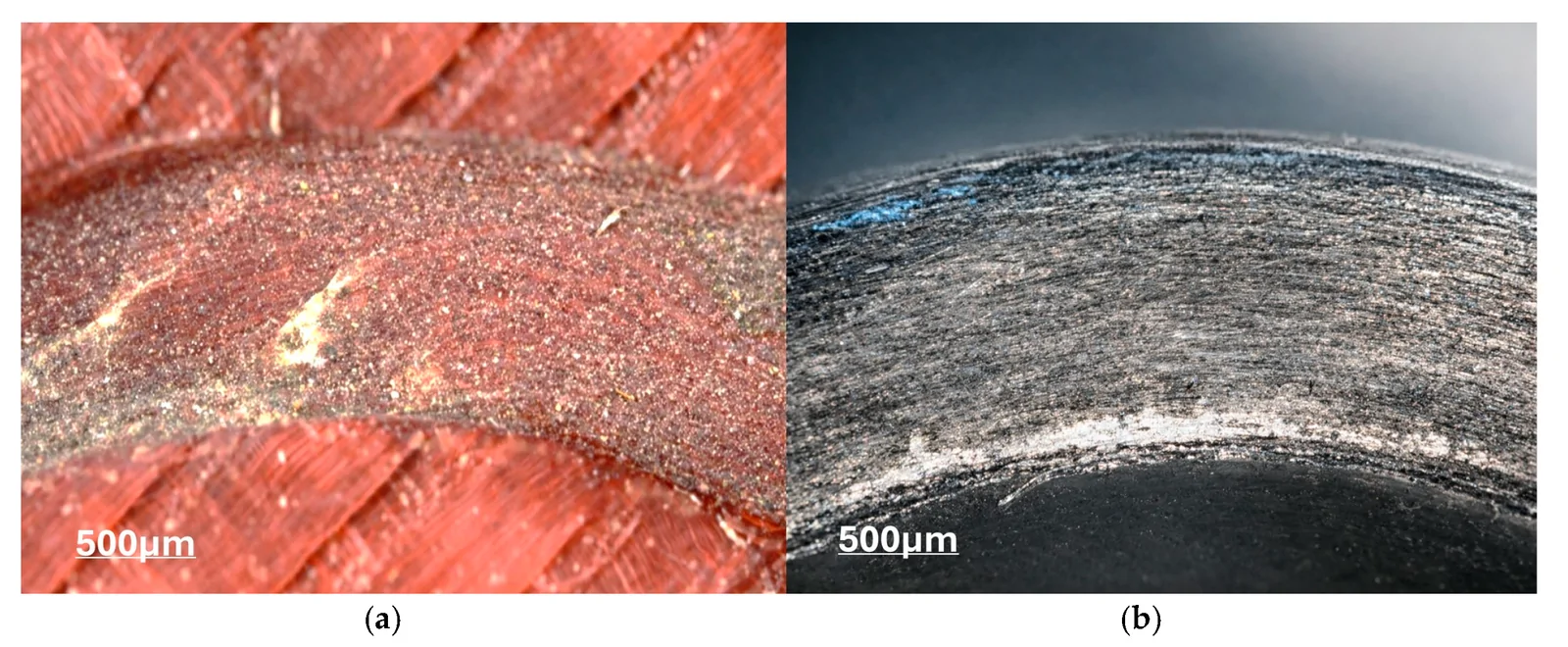
Catastrophic destruction of GFRP material from FP-520 (**a**) and AlCu4Mg2 alloy (**b**) during fretting corrosion tests.

Analysis of friction surfaces showed that when friction of the CFRP/GFRP-AlCu4Mg2 contact is rubbed under vibration load, rapid oxidation of the aluminum alloy occurs with the formation of Al_2_O_3_ oxides, which are known for their hardness and abrasive action [36]. Aluminum oxides with a hardness of 13.7 GPa and a compressive strength of 2 GPa, which is 95% of the hardness of diamond, destroy everything during reciprocating movement of samples under vibrations. Both composite materials and the corresponding aluminum alloy are subject to destruction (Figure 5).

The formation of Al_2_O_3_ is attributed to the fact that, in its initial state, the aluminum alloy surface bears only a thin (a few nanometers) native oxide film. During the reciprocating microslip of the composite materials, this film is rapidly destroyed and removed, exposing nascent metal surfaces. Aluminum has a very high affinity for oxygen; consequently, these exposed areas oxidize instantly upon contact with air, a process accelerated by local heating at actual contact points and by tribochemical reactions. Due to the small oscillation amplitude, oxide particles cannot escape the contact zone; instead, they accumulate there as a hard, abrasive “third body,” causing abrasive wear on both materials in the contact pair.

If we compare the wear resistance of CFRP and GFRP with almost the same epoxy binder, then the wear resistance of carbon fiber is almost two times greater than fiberglass. Carbon fibers, when destroyed by vibration, do not create an abrasive material, as fiberglass does. On the contrary, a dispersed graphite powder is created, which works as a solid lubricating layer. Such dependence can be traced at all contacts of test materials: CFRP-Al, CFRP-Fe, and CFRP-Ti.

**Figure 6:**
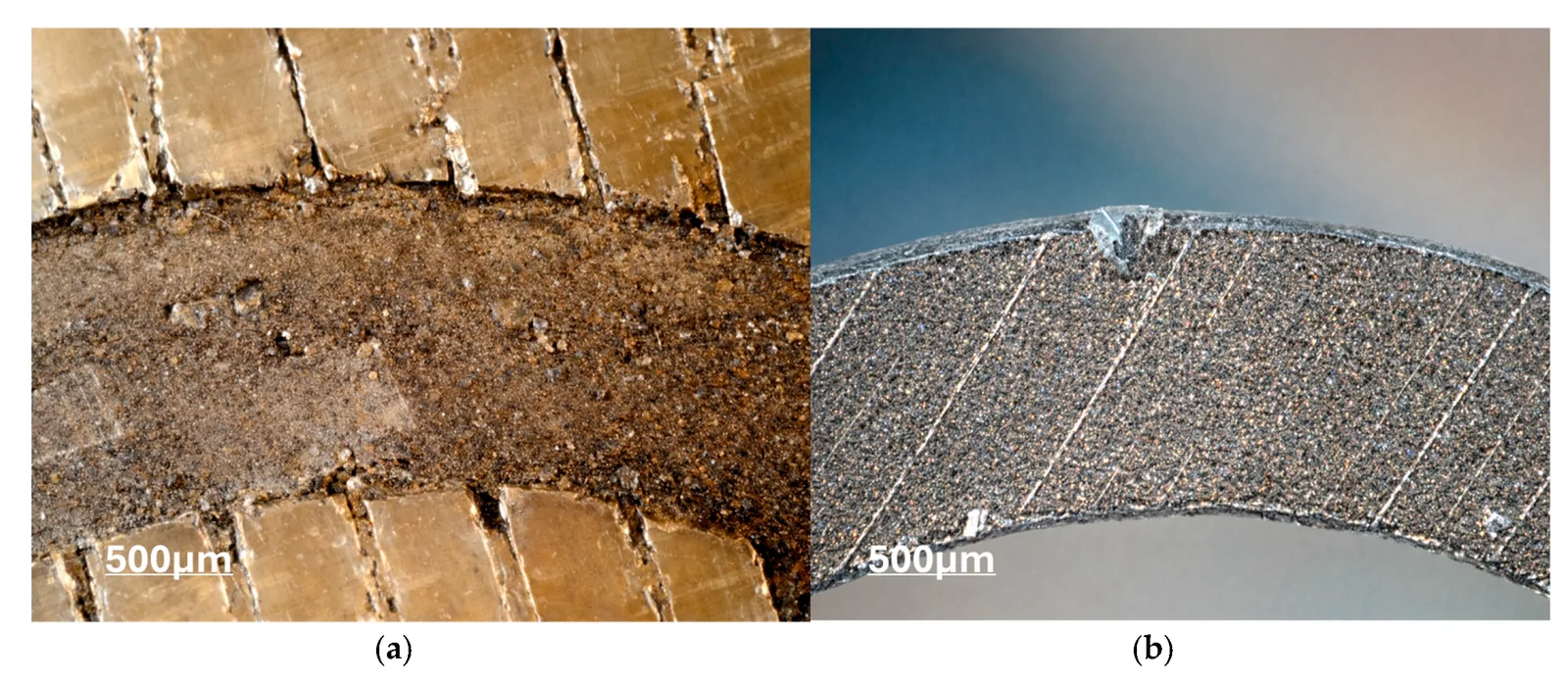
Topography of the GFRP surface with EDT-69H (**a**) and with 30CrMnSiA (**b**) steel during fretting corrosion tests.

Tests on the contact interaction of CFRP/GFRP with 30CrMnSiA steel revealed that the wear resistance of the composite materials is 2–4 times higher (Figure 4) than when in contact with other aviation alloys. This trend holds true for both CFRP and GFRP, as well as for materials with different types of binders. Contact between the CFRP/GFRP and 30CrMnSiA pair results in the formation of reddish-brown iron oxide (Fe_2_O_3_) on the surface; in terms of physicochemical properties, this oxide is significantly softer (500–600 HV) than Al_2_O_3_ (1500–2000 HV). Consequently, the rate of wear on the CFRP/GFRP surface caused by the steel under vibrational loading is significantly lower (Figure 6).

The formation of Fe_2_O_3_ is due to the fact that during fretting corrosion processes, the protective oxide film on the surface of 30KhGSA steel is destroyed, and new clean areas of iron are exposed to the air. These areas rapidly react with oxygen, a process accelerated by localized frictional heating and reaction dynamics at the contact interface. As a result, iron oxides are formed, which, upon further oxidation in the presence of atmospheric oxygen, transform into the most stable form Fe_2_O_3_, which explains the red-brown color of the wear products.

If we compare the wear of CFRP/GFRP, the results show a decrease in the wear of CFRP by 1.5 times compared to GFRP. When comparing the oxides of Al_2_O_3_ and Fe_2_O_3_ and their effect on the tribological characteristics of materials, the destructive effect of the abrasive Al_2_O_3_ in comparison with Fe_2_O_3_ during friction in the free state and an increase in the tribological characteristics of coatings when Al_2_O_3_ is added to it instead of iron oxides were established.

When analyzing the wear of CFRP/GFRP with titanium alloy Ti5Al5V5Mo1Cr1Fe, it can be said that this is the most successful combination among the tested alloys (Al, Fe, Ti). High adhesion susceptibility, lower surface strength than 30CrMnSiA steel and no abrasiveness of titanium oxides (TiO2) formed on the friction surface contribute to a reduction in the wear of CFRP/GFRP compared to aluminum alloy AlCu4Mg2 by 3–10 times and with steel 30CrMnSiA by 1.5–3 times, respectively (Figure 4). The pattern can be traced in tests on different types of CFRP/GFRP and different types of binder.

**Figure 7:**
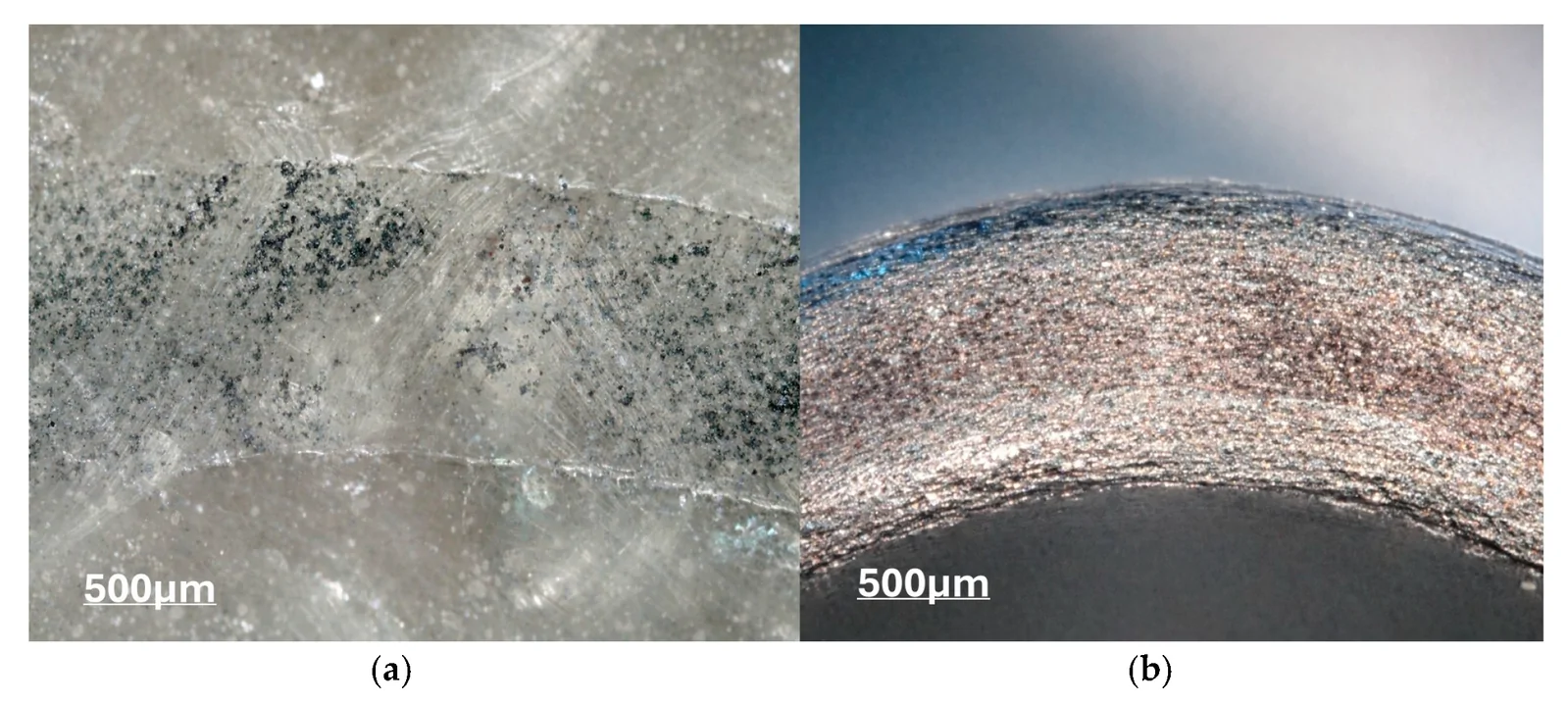
Topography of the GFRP surface with EDT-69H (**a**) and titanium alloy Ti5Al5V5Mo1Cr1Fe (**b**) during fretting corrosion tests.

**Figure 8:**
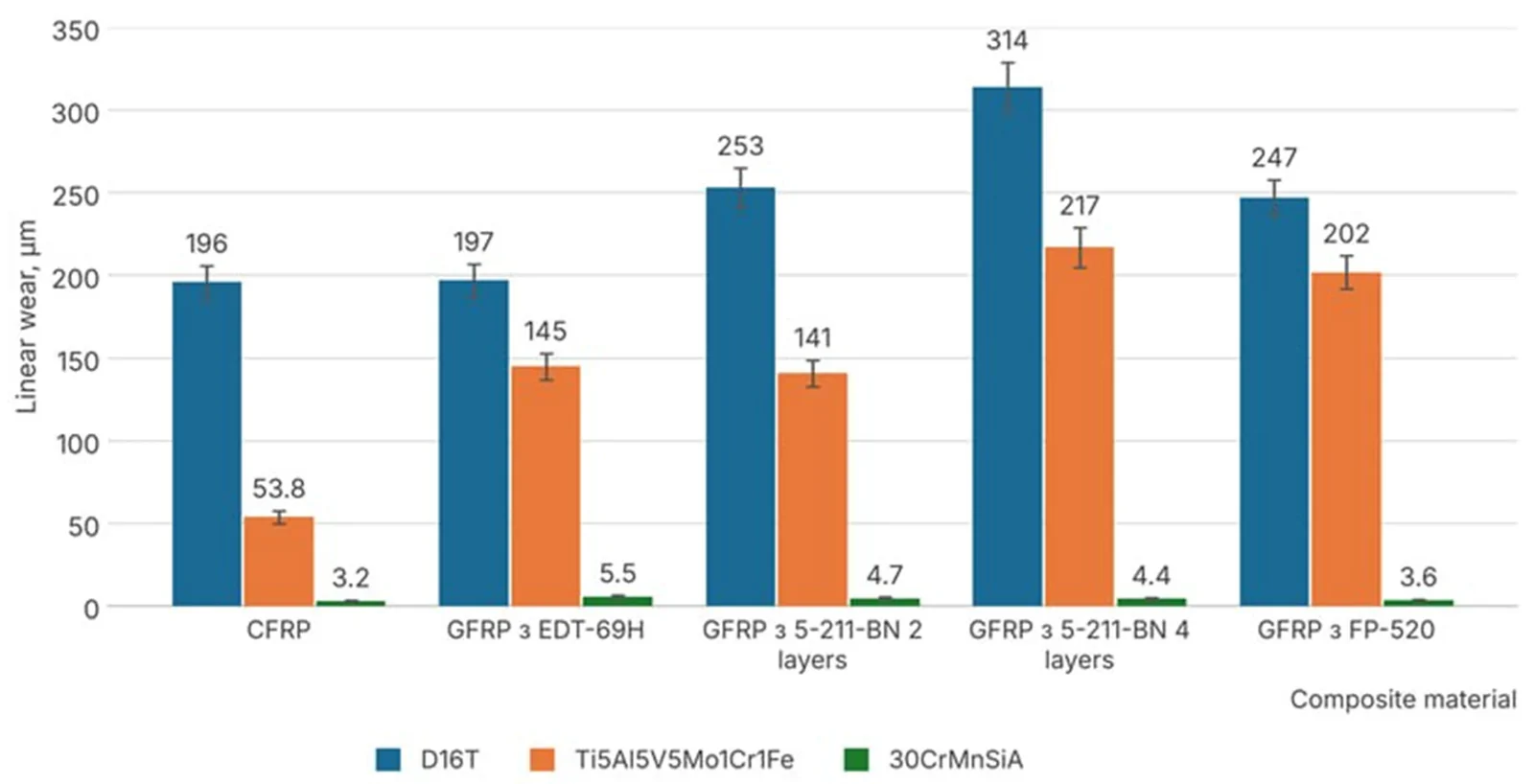
Wear resistance of aviation metal alloys with different types of CFRP/GFRP when tested under fretting corrosion conditions.

When comparing the wear of CFRP/GFRP paired with titanium alloy Ti5Al5V5Mo1Cr1Fe, we see a decrease in frictional wear with GFRP with EDT-69H by 10% compared to the wear of CFRP with ED-20 with almost identical epoxy matrices. The reason for the decrease in GFRP wear may be a higher surface strength than that of carbon fiber, as well as a difference in the physical and mechanical characteristics of the binder, which, although they have the same nature (epoxy resins), also differ in their physical and mechanical characteristics (Figure 7). When the titanium alloy is rubbed against a material with a higher surface strength, there is a greater activation of the areas of setting and transfer of the material to the surface of the composite material from the surface of the titanium alloy, which can cause an increase in the wear result of CFRP, which has a lower surface strength than fiberglass. At the same time, the destruction of the surface of the titanium alloy is many times faster than that of the composite material (Figure 8). This trend can be traced in all variants of contact between CFRP/GFRP and the titanium alloy Ti5Al5V5Mo1Cr1Fe.

**Figure 9:**
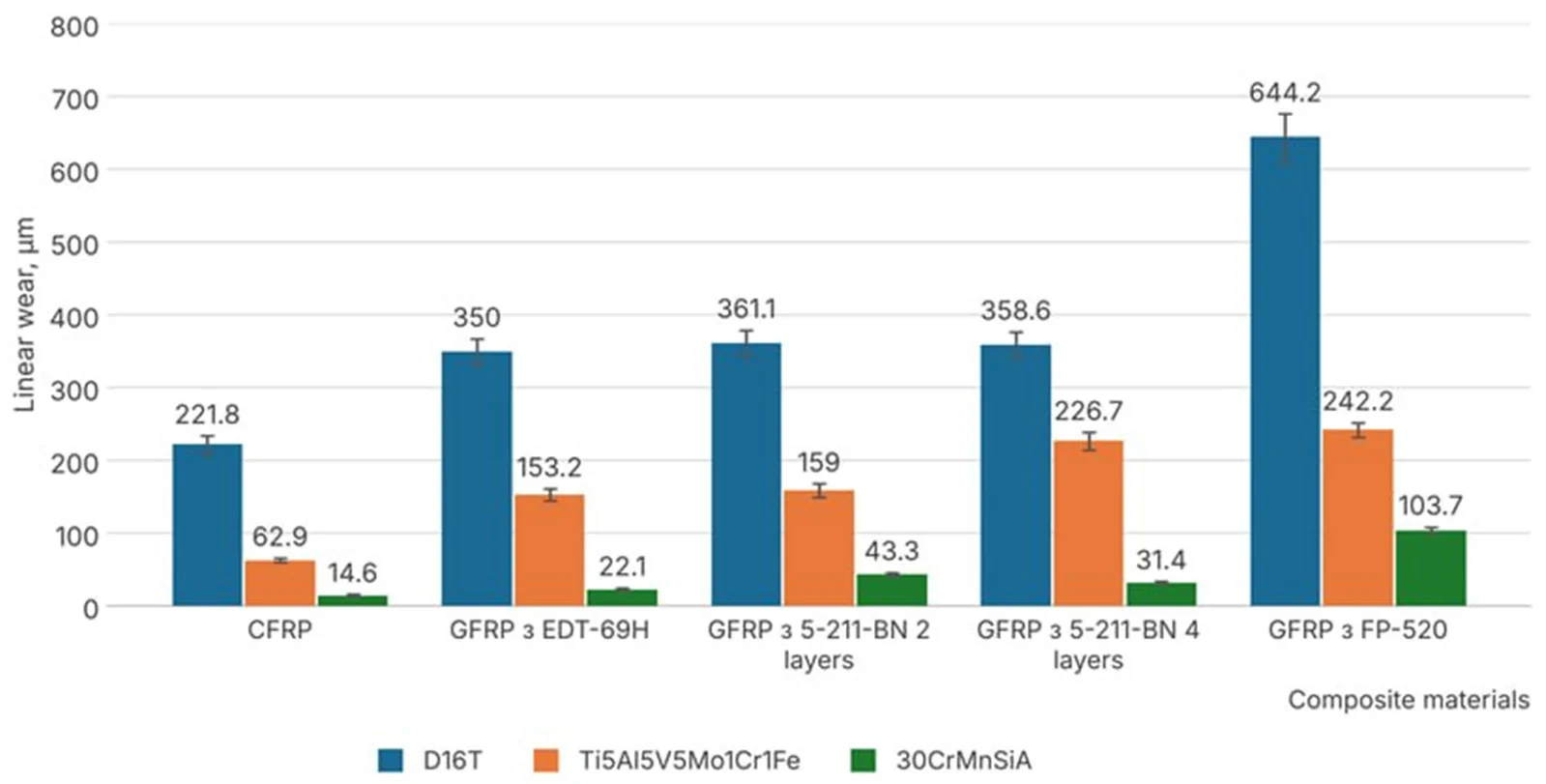
Total wear of CFRP/GFRP with aviation metal alloys during tests under fretting corrosion conditions.

Figure 8 presents the results of wear of metal aviation alloys in pairs with CFRP/GFRP, and Figure 9 presents the total wear of friction pairs, which characterizes the wear resistance of the contact pair as a whole and, as a consequence, the durability of the connection of the structure with these materials.

Analyzing the results of wear of aviation metal alloys paired with CFRP/GFRP, it can be concluded that the aluminum alloy AlCu4Mg2 has the greatest wear. When comparing the wear and tear of composite materials (Figure 4) with this material, we see that both CFRP/GFRP and the corresponding material AlCu4Mg2 are subject to catastrophic damage.

The contact of CFRP/GFRP with 30CrMnSiA steel is the most wear-resistant among the experiments. The wear resistance of both 30CrMnSiA steel samples in frustration corrosion tests and total wear of contact surfaces is the highest among the materials studied. The wear of steel samples is ten times less than the wear of CFRP/GFRP. The total wear of the Fe-CFRP/GFRP friction pair is 6–15 times less than the wear of the Al-CFRP/GFRP friction pair and 2.5–7 times less than the Ti-CFRP/GFRP contact.

## 4. Discussion

The increased tribotechnical properties of composite materials based on epoxy resins are also confirmed by a number of works. In Reference [37], the author studied the wear resistance of epoxy polymers and found that when modifiers and reinforcing fillers are added to the matrix, it allows for obtaining materials with stable physical and mechanical characteristics, and the use of functional additives allows for the achievement of the appropriate tribotechnical characteristics at the final stage of their creation of plain bearings, which increases wear resistance by 1.5–2.0 times. The authors of References [37,38] found that the wear resistance of industrial epoxy composite materials using silicon carbide is not inferior to world analogs in terms of wear resistance and deformation parameters under contact-dynamic loading. It has been determined that the behavior of filled wear-resistant epoxy composite materials during contact-dynamic deformation is determined by the features of their structure, which affects the time of the main phases of the process, the components of deformation energy and their ratio, and, as a result, the wear resistance of materials.

The formation of abrasive material by friction with aluminum alloys under condi-tions of fretting corrosion is also mentioned in References [36,39]. It is thanks to this phe-nomenon that aluminum alloy is not used in its pure form in aviation structures. There is always a protective coating or surface treatment of the alloy. This is especially true when aluminum alloy comes into contact with CFRP/GFRP, where it is strictly prohibited by the technical documentation to use aluminum alloy without processing.

The effect of the corrosive effect of fretting corrosion enhances humidity and sea air when aircraft fly near the coasts of the seas and oceans. The analysis of damage to the contact of CFRP/GFRP with aviation materials for An-26, An-32, An-32P, An-74, Mi-8MTV, Mi-171, Boeing 737, etc. showed the catastrophic destruction of the points of contact of composite materials with aluminum alloy in the humid environment of the African continent in the conditions of vibration load, due to the enhanced effect of electrochemical corrosion.

In this case, the technical documentation provides for the protection of the aluminum alloy by surface anodizing (the oxide film does not conduct electric current) or the use of protective paints and varnishes (primer) at the contact of CFRP/GFRP-Al. The destruction of aluminum aircraft parts is enhanced by contact with carbon fiber. Moist sea air quickly destroys the surface of the aluminum alloy due to the electrical conductivity of carbon fibers. In this case, for example, in Boeing 737 aircraft, at the junction of the skin with carbon fiber, spacers of titanium sheets are placed between the Al-CFRP contact to avoid the destructive effect of electrochemical corrosion.

**Figure 10:**
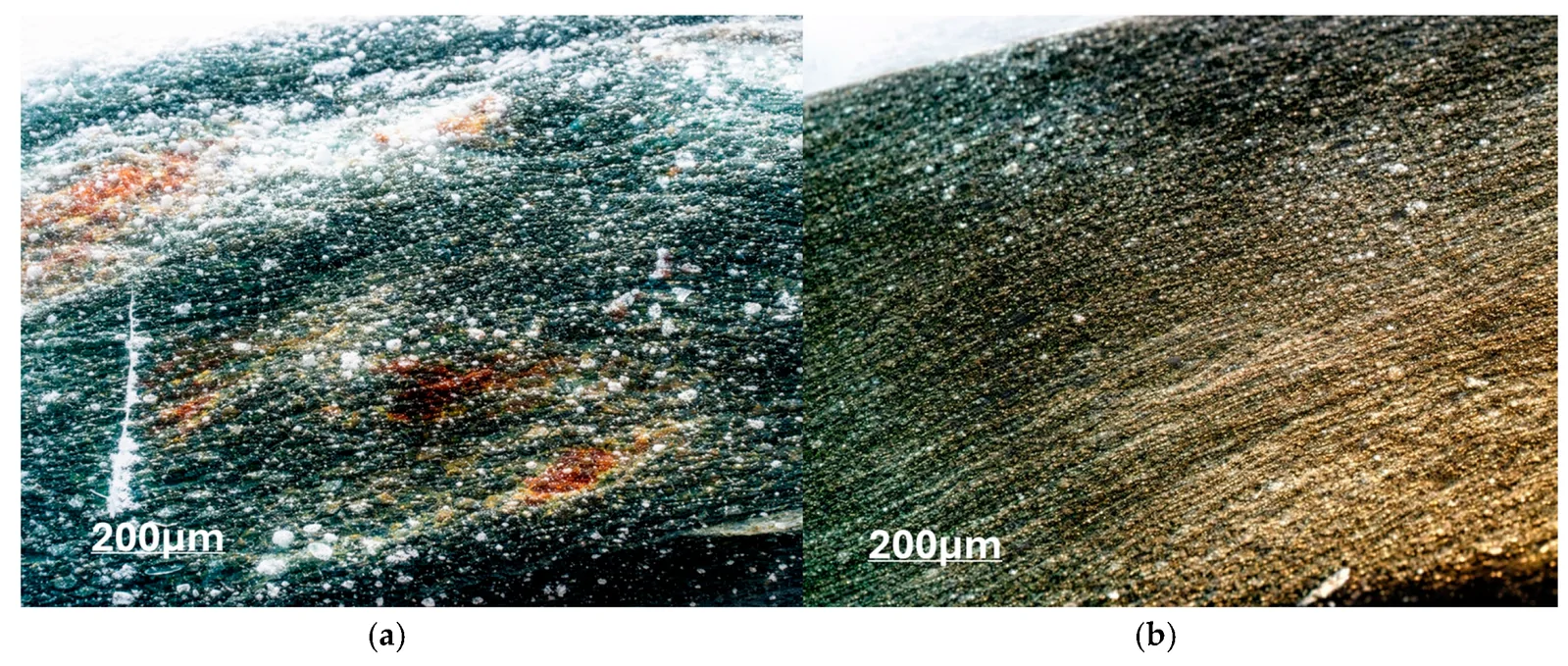
Topography of friction surfaces of aluminum alloy AlCu4Mg2 (**a**) and titanium alloy Ti5Al5V5Mo1Cr1Fe (**b**) after fretting corrosion tests paired with CFRP.

The wear of the aluminum alloy AlCu4Mg2 in comparison with CFRP/GFRP is 2–7 times greater. The suspended surface strength of CFRP/GFRP and the abrasive material Al_2_O_3_ formed in the process of vibration loads destroy the surfaces of the contacting materials at a catastrophic rate, which confirms the total wear of the contact pair, which is shown in Figure 9. The total wear of the Al-CFRP/GFRP contact pair is 1.5–3 times greater than the wear of the Ti-CFRP/GFRP contact and 5–11 times greater than the Fe-CFRP/GFRP contact. That is why the contact of aluminum alloy with composite materials in modern aircraft types is massively undergoing attempts to be replaced with a contact of Ti-CFRP/GFRP, the total wear of which is less, and the damage to the surfaces is more uniform (Figure 10). This is the path taken by such global manufacturers as Boeing and Airbus when designing their aircraft with a large amount of CFRP/GFRP. If it is impossible to replace the aluminum alloy in contact with CFRP/GFRP with another metal, then according to [40], one of the following options is used:

1. Anodizing aluminum alloy coating (to increase the wear resistance of the aluminum surface to prevent electrical conductivity and corrosion);
2. Coating of aluminum alloy with polymer films (prevention of electrical conductivity and prevention of fretting corrosion);
3. Application of titanium plates in contact between aluminum alloy and CFRP (prevention of corrosion from moisture and sea air);
4. Use of aluminum special alloys (use of soft aluminum alloys with increased properties of corrosion and increased fretting resistance);
5. Use of tight contacts (to prevent the occurrence of fretting corrosion by reducing the amplitude of mutual displacement in the contact to zero or very small values).

The contact of high-strength steel 30CrMnSiA with CFRP/GFRP is quite popular on aircraft of the aging fleet, which were designed in the 70–80s of the previous century. It resists vibration loads quite well and does not cause catastrophic destruction in composite materials based on carbon fiber and glass fiber. However, in pursuit of improving fuel and economic efficiency, according to the latest environmental performance regulations ISO 14001:2015 [41] and 19011:2018 [42], in modern aircraft, lighter materials are used. It is titanium alloy that is very often used as a replacement for steel structures. Along with titanium alloy, aluminum alloys are also used, but with certain limitations mentioned above.

The Ti-CFRP/GFRP contact is the most promising from the point of view of economic efficiency and increased needs for the use of composite materials in modern aircraft. For the next 10–50 years, this contact will be the most prevalent in aircraft and aircraft engines, where it is physically impossible to obtain a CFRP-CFRP contact.

The wear resistance of titanium alloy Ti5Al5V5Mo1Cr1Fe during tests with CFRP/GFRP showed indirect results under vibration load conditions. Analyzing the wear resistance of titanium samples made of CFRP/GFRP, it can be said that the wear of the alloy Ti5Al5V5Mo1Cr1Fe is from six to 20 times higher than the wear of CFRP/GFRP, which is confirmed by the results of wear (Figure 8). The wear resistance of titanium samples in comparison with aluminum alloy is 1.5–3 times higher, and it seems that titanium alloy does not behave much better than aluminum alloy under vibration loads. However, having analyzed the total wear of the friction pair (Figure 9), we can say that in comparison with the Al-CFRP/GFRP contact, the total wear resistance of contact with titanium with the Ti-CFRP/GFRP alloy is 2–5 times higher.

Increased wear resistance characteristics of titanium alloys compared to aluminum alloys and increased resistance to corrosion of humid and marine environments make it possible to use the Ti-CFRP/GFRP contact in modern high-performance aircraft. Another advantage of titanium alloys in comparison with aluminum alloys is the possibility of technological methods for strengthening the surfaces of titanium alloys [43,44] when they come into contact with CFRP/GFRP. Modern technological methods make it possible to change the surface strength of titanium alloys up to 1000 times, and as a result, the wear resistance of titanium alloy parts under vibration load.

**Figure 11:**
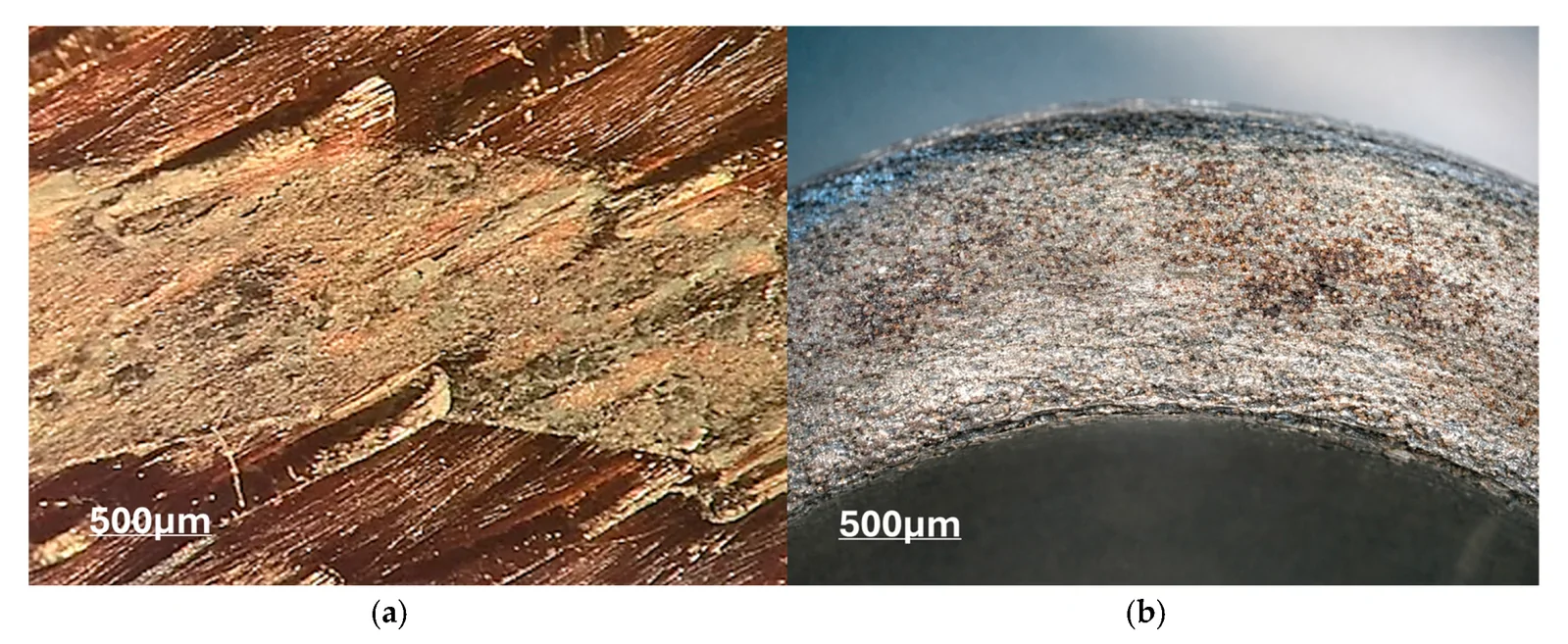
Topography of the surface of CFRP (**a**) and titanium alloy Ti5Al5V5Mo1Cr1Fe (**b**) during fretting corrosion tests.

Analyzing the total wear resistance between CFRP and GFRP with an epoxy-based binder, a higher wear resistance is observed in both CFRP samples and aviation metal alloys compared to GFRP. That is, the global trend in choosing the CFRP-Ti contact for structural elements in modern aircraft is confirmed by studies of wear resistance in conditionally stationary contact [45]. The topography of friction surfaces of CFRP (Figure 11) with titanium alloy during wear resistance tests under fretting corrosion conditions does not differ in relief with GFRP. The friction surface of composite materials after removal of wear products from the surface is smooth with a uniform relief, which allows for the use of carbon fibers even in places where micro movements occur between contacting materials. The analysis did not reveal the occurrence of microcracks in the materials of samples with CFRP/GFRP after testing. The absence of microcracks was confirmed through microscopic examination of the specimens’ friction surfaces following the removal of wear debris; inspections were conducted using an optical microscope at magnifications ranging from ×50 to ×500, a range capable of detecting sub-micrometer cracks. Additionally, cross-sections of the specimens in the contact zone were analyzed; no cracks, fiber–matrix delamination, or fiber breakage were observed at depths beneath the wear scar. The absence of defects is further evidenced by the surface’s uniform topography, which lacks the localized pitting or spalling typically associated with crack propagation. Consequently, during fretting against a titanium alloy, wear in these composites occurs through the gradual abrasion of the surface layer rather than fatigue cracking, making them suitable for applications involving micro-displacements. A feature of the application may be the radio transparency and dielectric properties of GFRP and the non-radio transparency and electrical conductivity of CFRP, which is a limitation, for example, in the use of military drones.

However, if we compare the wear resistance of titanium alloy with the wear resistance of composite materials, then a question arises with regard to protecting the surfaces of parts made of titanium alloys, rather than protecting CFRP/GFRP materials, since the wear resistance of CFRP/GFRP is 6–20 times higher than that of titanium alloy under conditions of nominally stationary contact under vibration loads.

Thus, given that the Ti-CFRP/GFRP contact is the most widespread in modern aircraft construction and in the coming decades, it is necessary to investigate in more detail the wear resistance of the contact of these materials under vibration load in a wide range and the possibility of protecting titanium parts with special technological methods to increase wear resistance.

## 5. Conclusions

Thus, having analyzed the study of wear resistances of various types of composite materials with different types of binder, paired with aviation metal alloys, the following conclusions can be drawn:

1. The contact of Al-CFRP/GFRP leads to catastrophic destruction of friction surfaces, both aluminum alloy (Figure 10) and composite materials, due to the formation of solid oxides of Al_2_O_3_ under vibration load. This type of contact should always be protected by additional methods of surface treatment of aluminum alloys to prevent oxidation. Destruction enhances moist and marine air, as well as electrochemical corrosive reactions in contact.
2. The best contacts in terms of wear resistance with CFRP/GFRP under vibration loads are steel. The total wear resistance is the lowest among the test contact options. However, in modern aircraft, attempts are being made to replace this contact with the Ti-CFRP/GFRP contact, to increase fuel efficiency and, as a result, economic efficiency.
3. The wear resistance of CFRP is higher compared to GFRP in the study of aviation materials under vibration loads, due to the formation of solid abrasive particles of glass fibers on the GFRP surface and their abrasive action. In the case of CFRP, carbon dust is formed on the surface, which helps to reduce friction in contact and works as a solid lubricant.
4. The contact Ti-CFRP/GFRP is the most promising for modern aircraft. The wear resistance of composite materials is 6–20 times higher than that of titanium alloy due to the nature of titanium and its tendency to set. Aircraft structures made of CFRP/GFRP in contact with titanium alloys have sufficient wear resistance under vibration loads and can be used to a limited extent without special surface treatment cracks. Due to the high wear of titanium alloy paired with composite materials, it must be used with protective coatings and technological methods to increase wear-resistant characteristics.

## Data Availability

The original contributions presented in this study are included in the article. Further inquiries can be directed to the corresponding authors.
